# Evaluación de un protocolo de centrifugación alternativo que permita reducir el tiempo de respuesta total

**DOI:** 10.1515/almed-2024-0119

**Published:** 2024-11-11

**Authors:** Reyes Nicolás de Blas, Miriam Menacho Román, Sara Olivares Romero, Claudia Mesas Mariñán, Alba Arroyo Vega, Graciela Martín Gómez, María Álvarez Pastor, Lucía Castillo Menéndez, María José Azofra Villa, María del Pilar Pérez Sobrino, Ignacio Arribas Gómez

**Affiliations:** Servicio De Bioquímica Clínica, Hospital Universitario Ramón Y Cajal, Madrid, España; Extracciones Centrales, Hospital Universitario Ramón Y Cajal, Madrid, España

**Keywords:** centrifugación, tiempo de respuesta, preanalítica

## Abstract

**Objetivos:**

La centrifugación es un proceso clave que debemos controlar para asegurar una adecuada calidad de la muestra. Con el objetivo de conseguir una centrifugación de muestras unificada y estandarizada en el menor tiempo posible, nos propusimos evaluar un protocolo de centrifugación alternativo y su influencia en los resultados de 20 determinaciones bioquímicas en suero.

**Métodos:**

Fueron incluidos en el estudio 45 pacientes ambulatorios. A cada paciente se le extrajeron 2 tubos de suero con gel separador (*Becton Dickinson (BD) Vacutainer* ^*®*^ *SST™ II Advance, 8,5 mL Ref. 366468*)*.* Uno de ellos fue centrifugado a 2530×*g* durante 10 minutos, método control. El otro tubo de suero fue centrifugado en unas condiciones de centrifugación alternativas, a 2530×*g* durante 7 min.

**Resultados:**

El análisis de los resultados muestra que el calcio, la bilirrubina total y el magnesio presentan un error sistemático constante y proporcional. Sin embargo, atendiendo a la diferencia de medias proporcional, todas las magnitudes cumplen el requisito del error sistemático deseable aceptado por nuestro laboratorio, a excepción del magnesio, que cumple el error sistemático mínimo.

**Conclusiones:**

Nuestro estudio demuestra que ambas condiciones de centrifugación son intercambiables para la medición de las magnitudes estudiadas, asegurando una adecuada calidad de la muestra.

## Introducción

La centrifugación es un procedimiento preanalítico que se realiza innumerables veces al día en los laboratorios clínicos, sin embargo, son pocos los estudios que documentan la influencia de la centrifugación en los resultados de laboratorio [1], [2], [3], [4], [5], [6]. Se trata de un proceso clave que debemos controlar para asegurar una adecuada calidad de la muestra.

Cada laboratorio debe establecer un protocolo estandarizado de centrifugación. Se debe definir el RCF (*relative centrifugal force*), temperatura y tiempo óptimos para obtener muestras de alta calidad para cada tipo de contenedor, basándose en las recomendaciones del fabricante y los requerimientos de tiempo de respuesta (*turnaround time,* TAT). Cualquier desviación de estas recomendaciones debe ser validada [7].

Es de crucial importancia en la calidad del laboratorio clínico un adecuado TAT. El laboratorio clínico debe ofrecer un informe rápido, fiable y exacto, que permita una toma de decisiones clínicas adecuadas y en tiempo. El TAT es considerado por los programas de garantía de calidad como un indicador de eficacia de los laboratorios, siendo imprescindible su medición sistemática y análisis para garantizar la calidad extraanalítica. Los plazos de entrega de resultados deben ser registrados y revisados para tomar acciones correctivas en caso de identificar problemas [8].

Las recomendaciones generales de centrifugación para los parámetros bioquímicos analizados en suero son de 1000-3000×*g* durante 10–15 minutos a temperatura entre 15-24 °C [7]. Existen procedimientos alternativos de centrifugación aceptados que implican una mayor fuerza g (RCF) y un menor tiempo de centrifugación, consiguiendo con ello una disminución del TAT [7].

Las condiciones de centrifugación alternativas validadas por Becton Dickinson (BD) en el estudio *BD White Paper* VS*7228* [9], demuestran equivalencia entre la condición control (1300×*g*-10 minutos) y la condición alternativa evaluada (3000×*g*-5 min). En nuestro caso no es posible la centrifugación a 3000×*g* por recomendación del fabricante (Kubota S700TR), por lo que se propone una centrifugación a 2530×*g* (máxima velocidad disponible) a 7 min [9].

Nuestro objetivo es conseguir una centrifugación de muestras unificada y estandarizada con el menor tiempo posible para todos los tipos de muestra sin alterar de manera significativa la calidad del plasma/suero. Como primera aproximación se realiza el estudio únicamente con las muestras de suero para la determinación de parámetros bioquímicos.

En este estudio se pretende evaluar el rendimiento clínico de los tubos *BD Vacutainer* ^*®*^ *SST*™ *II Advance* con las siguientes condiciones de centrifugación: velocidad de 2530×*g* y un tiempo de 7 min.

Se considera como condición control de centrifugación 2530×*g* de velocidad y 10 minutos de tiempo.

## Materiales y métodos

El estudio se llevó a cabo en el Servicio de Bioquímica clínica del Hospital Universitario Ramón y Cajal (HURYC) durante los meses de mayo y junio de 2024.

El proyecto fue aprobado por el Comité de Ética del HURYC con código 046/24.

Fueron incluidos en el estudio 45 pacientes ambulatorios con edad comprendida entre 18 y 70 años, de ambos sexos y que aceptaron participar firmando un modelo de consentimiento informado. Los pacientes oncológicos fueron excluidos.

A cada paciente se le extrajeron 2 tubos de suero con gel separador *(BD Vacutainer* ^*®*^ *SST*™ *II Advance, 8,5 mL Ref. 366468).* La extracción se realizó utilizando un dispositivo de palomilla de seguridad Vacuette 21G (0,80× 19 mm) con portatubos premontado *(Ref. 450085V1 Greiner Bio-One*) y siguiendo las recomendaciones de la guía *Clinical and Laboratory Standards Institute* (CLSI) GP 41 [10] y del documento elaborado conjuntamente por la *European Federation of Clinical Chemistry and Laboratory Medicine* (EFLM) y la *Latin America Confederation of Clinical Biochemistry* [11].

El tratamiento de las muestras previo al análisis (extracción, transporte, tiempo y temperatura) fue el mismo para ambos pares de muestras, limitando así la presencia de otras variables en el estudio.

Las muestras fueron almacenadas a temperatura ambiente en posición vertical un tiempo mínimo de 30 min, asegurando la retracción total del coágulo.

Las muestras fueron centrifugadas en una única centrífuga, modelo Kubota S700TR de rotor basculante (Rotor RS-1440 M) (mantenimiento preventivo realizado previamente al comienzo del estudio). Una de las muestras de cada paciente fue centrifugada a 2530×*g* durante 10 min, método control. El otro tubo de suero fue centrifugado en unas condiciones de centrifugación alternativas, a 2530×*g* durante 7 min. Todas las muestras fueron centrifugadas a temperatura ambiente (18–22 °C). El orden de extracción de los tubos y el proceso de centrifugación de ambos grupos se llevó a cabo de forma aleatoria.

Se analizaron 20 determinaciones bioquímicas (Tabla 1) en un analizador Alinity c (Abbott Diagnostics, Illinois, Estados Unidos). Las muestras fueron procesadas por duplicado, inmediatamente después de la centrifugación.

### Análisis estadístico

Los datos *outliers* fueron desestimados siguiendo las recomendaciones de la guía EP09-A2 CLSI [12].

El estudio descriptivo de las variables cuantitativas se realizó mediante la media y la desviación estándar, en caso de una distribución normal, y mediante la mediana y los percentiles 25 y 75, en caso contrario, para cada condición de centrifugación.

Para comprobar el supuesto de normalidad de las variables se realiza el test no paramétrico Kolmogorov-Smirnov.

Una vez comprobada la relación lineal entre las series, se evalúa la concordancia entre ambos procedimientos mediante el diagrama de Bland-Altman [13] y el coeficiente de concordancia de Lin [14].

De acuerdo a las especificaciones de calidad del laboratorio, se admite como desviación máxima admisible, el error sistemático (ES) deseable basado en variabilidad biológica (coeficientes de variación intra e interindividual, CVI y CVG), obtenido de la base de datos de la EFLM, de acuerdo a la siguiente fórmula [15]:

 ES<0,25 × (CVI^2^ + CVG^2^)^1/2^

Los cálculos se realizaron con MedCalc^®^ 12.3 (MedCalc Software, Ostende, Bélgica). Se ha considerado significativo un valor de p<0,05.

## Resultados

Las determinaciones de ácido úrico y triglicéridos tuvieron que desestimarse al superar el número de puntos aberrantes admisibles para la comparación. Para todos los demás parámetros, se obtuvieron valores adecuados en un mínimo de 44 sujetos (Tabla 1).

**Table 1:** Comparación de los resultados obtenidos entre las condiciones de centrifugación estudiadas. Estudio descriptivo.

Analito	Unidades	n	Rango de estudio	Media ± DE/mediana [p25–p75]	
				2530 ×*g* 10 min.	2530 ×*g* 7 min
Glucosa	mg/dL	44	72–142	95,66 ± 14,27	95,90 ± 14,47
Sodio	mmol/L	45	136–145	140,59 ± 2,02	140,62 ± 2,15
Potasio	mmol/L	45	3,71–6,57	4,54 ± 0,47	4,51 ± 0,45
Cloro	mmol/L	45	100–114	106,44 ± 2,89	106,31 ± 2,97
Creatinina	mg/dL	45	0,53–2,82	1,02 ± 0,45	1,02 ± 0,45
Fósforo	mg/dL	44	2,29–4,63	3,37 ± 0,59	3,36 ± 0,59
Calcio	mg/dL	45	8,5–10,9	9,60 ± 0,48	9,64 ± 0,50
Colesterol	mg/dL	45	107–256	178,74 ± 37,74	178,49 ± 37,41
Proteínas totales	g/dL	45	6,25–8,05	7,08 ± 0,37	7,08 ± 0,36
Bilirrubina total	mg/dL	45	0,24–2,69	0,66 ± 0,40	0,66 ± 0,40
Fosfatasa alcalina	U/L	45	36–173	81,59 ± 72,78	81,28 ± 27,74
Alanina aminotransferasa	U/L	44	7–50	20,56 ± 9,67	20,55 ± 9,65
Lactado deshidrogenasa	U/L	45	92–317	187,94 ± 37,88	188,21 ± 39,29
Hierro	µg/dL	45	28–159	88,30 ± 31,79	88,31 ± 31,86
Magnesio	mg/dL	45	1,53–2,34	1,94 ± 0,16	1,97 ± 0,15
Urea	mg/dL	44	18–126	33,25 [28,50 a 45,75]	33,00 [28,50 a 46,25]
Gamma-glutamil transferasa	U/L	45	11–247	25,00 [20,37 a 36,62]	25,5 [20,00 a 36,00]
Aspartato aminotransferasa	U/L	45	11–51	20,00 [17,87 a 23,12]	21,00 [17,87 a 23,50]

DE, desviación estándar; p25, percentil 25; p75, percentil 75.

El análisis de los resultados mediante las pruebas de concordancia de Bland-Altman muestran que el calcio, la bilirrubina total y el magnesio presentan un error sistemático constante y proporcional, al no incluir el intervalo de confianza de las diferencias absolutas y relativas el valor 0. Sin embargo, atendiendo a la diferencia de medias proporcional, todas las magnitudes, a excepción del magnesio, cumplen el requisito del error sistemático deseable aceptado por nuestro laboratorio.

**Figure 1:**
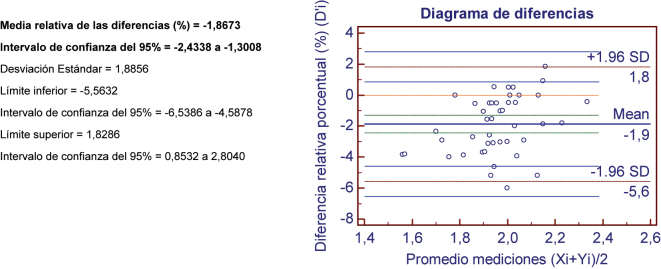
Se muestra los resultados del estudio de concordancia de Bland-Altman en el estudio del magnesio a modo de ejemplo. Yi: promedio de los resultados del magnesio en el método de evaluación (7 min). Xi: promedio de los resultados del magnesio en el método control (10 min). D’i: Diferencia relativa porcentual D’i: Xi-Yi/[(Xi + Yi)/2]*100. Los datos son comentados en el texto.

Como se muestra en la Figura 1, el método de evaluación para la determinación del magnesio presenta sistemáticamente valores ligeramente más elevados que el método control. Esta diferencia se mantiene estable en todas las concentraciones de estudio. Sin embargo, atendiendo a criterios biológicos, pese a no cumplir el requisito de error sistemático deseable, el valor de las diferencias relativas del magnesio es inferior al error sistemático mínimo propuesto por la EFLM [15].

Los resultados de todas las magnitudes de estudio se describen en las Tablas 1 and 2.

**Table 2:** Comparación de los resultados obtenidos entre las condiciones de centrifugación estudiadas. Estudio de concordancia.

Analito	Bland-Altman	Límite de aceptación, %	Coeficiente de concordancia de Lin
	Media relativa de las diferencias (IC 95 %)		
Glucosa	0,26 (−0,18 a 0,70)	1,2	0,9985
Sodio	−0,03 (−0,17 a 0,13)	0,1	0,9421
Potasio	0,43 (−0,64 a 1,51)	0,8	0,9358
Cloro	−0,13 (−0,26 a 0,01)	0,2	0,9855
Creatinina	0,45 (−0,12 a 1,01)	2,1	0,9991
Fósforo	0,22 (−0,23 a 0,66)	1,6	0,9970
Calcio	**−0,42 (-0,65 a -0,19)**	**0,8**	0,9847
Colesterol	0,11 (−0,20 a 0,43)	2	0,9987
Proteínas totales	−0,02 (−0,35 a 0,30)	0,5	0,9774
Bilirrubina total	**−0,71 (-1,36 a -0,06)**	**4**	0,9995
Fosfatasa alcalina	0,40 (−0,01 a 0,81)	2,7	0,9991
Alanina aminotransferasa	0,23 (−0,74 a 1,20)	5	0,9984
Lactado deshidrogenasa	−0,07 (−1,55 a 1,41)	1,6	0,9626
Hierro	−0,02 (−0,59 a 0,56)	4,8	0,9985
Magnesio	**−1,87 (-2,43 a -1,30)**	**2,4**	0,9448
Urea	0,14 (−0,31 a 0,59)	3,1	0,9997
Gamma-glutamil transferasa	0,71 (−0,09 a 1,51)	5,7	0,9998
Aspartato aminotransferasa	−0,98 (−2,18 a 0,22)	2,7	0,9941

Se establece como límite de aceptación el error sistemático deseable (%), a excepción del magnesio (error sistemático mínimo)-base de datos EFLM. Coeficiente de concordancia de Lin: >0,99 Concordancia casi perfecta; 0,95–0,99 concordancia sustancial; 0,90–0,95 concordancia moderada; <0,90 concordancia pobre. IC, 95 %: intervalo de confianza de 95 %. En negrita se indican los resultados del calcio, bilirrubina total y magnesio, comentados en el texto.

## Discusión

Para garantizar un adecuado TAT es posible realizar ajustes de centrifugación, siempre y cuando no se comprometa la calidad de la muestra.

Siguiendo las recomendaciones validadas por BD [9], propusimos una evaluación de unas condiciones de centrifugación alternativas factibles en nuestro laboratorio, 2530×*g *a 7 min.

La mayoría de estudios previos han demostrado una precisión analítica equivalente utilizando tiempos y RCF alternativas con otros tubos de recogida e instrumentos [1], [2], [3], [4], [5]. En general, investigaciones previas demuestran que el tiempo de centrifugación puede reducirse por debajo de los 10 min sin afectar negativamente a la calidad de la muestra en la mayoría de los parámetros estudiados. Sin embargo, dada la gran disparidad en las condiciones de centrifugación recomendadas, los resultados de los estudios que utilizan un tubo concreto de recogida pueden no ser generalizables a los tubos de recogida de distintos fabricantes. Otros estudios demuestran una alteración de la calidad de la muestra con RCF superiores a 4000×*g* [6].

Consideramos conveniente estudiar adicionalmente otros aspectos relacionados con la calidad de la muestra no realizados en nuestro estudio, como son aspectos relacionados con la barrera de gel y el rendimiento sérico, evaluados por BD en el estudio *BD White Paper* VS*7228* [9], o la presencia de restos de fibrina o células residuales en el suero/plasma.

Cada laboratorio debe realizar una validación de sus propias condiciones de centrifugación si éstas varían de las propuestas por el fabricante de tubos y/o reactivos.

Centrifugamos diariamente cerca de 3000 muestras, de las que un 28 % requieren prioridad urgente, en aproximadamente 40 tandas de centrifugación. Teóricamente, una reducción de 3 minutos en este proceso supondría un ahorro de 120 minutos diarios. Se considera conveniente realizar un estudio posterior para confirmar la reducción del TAT en las condiciones reales de trabajo.

El elevado número de muestras que requieren centrifugación y la necesidad de obtener resultados en un corto periodo de tiempo, justifica la reducción de los 3 minutos en el proceso de centrifugación.

Podemos concluir que nuestro estudio demuestra que ambas condiciones de centrifugación son intercambiables para la medición de las magnitudes estudiadas, asegurando una adecuada calidad de la muestra.

Se requiere un estudio más extenso donde se incluyan el resto de parámetros y especímenes (plasma) para obtener conclusiones más robustas.
